# Frequency of Celiac Disease in Irritable Bowel Syndrome: Insights From a Tertiary Care Hospital

**DOI:** 10.7759/cureus.89617

**Published:** 2025-08-08

**Authors:** Muhammad Usman Tufail Warraich, Muhammad Nabeel Shafqat, Usman Ajmal, Muhammad Adil Choudhary, Ayesha Shaukat, Nabeel Saleem, Muhammad Affan-Ud-Din, Namra Khaliq, Adeel Yaseen, Hamza Riaz

**Affiliations:** 1 Gastroenterology and Hepatology, Queen Alexandra Hospital, Portsmouth, GBR; 2 Internal Medicine, Three Crosses Regional Hospital, Las Cruces, USA; 3 Gastroenterology and Hepatology, Gujranwala Medical College Teaching Hospital, Gujranwala, PAK; 4 Gastroenterology, University of Health Sciences Lahore, Gujranwala, PAK; 5 Gynecology, Kalsoom Tufail Hospital, Gujranwala, PAK; 6 Cardiology, Portsmouth Hospitals University NHS Trust, Portsmouth, GBR; 7 Medicine, Cavan General Hospital, Cavan, IRL; 8 Internal Medicine, Sadiq Hospital, Sargodha, PAK; 9 Internal Medicine, Mayo Hospital, Lahore, PAK

**Keywords:** anti-tissue transglutaminase antibody, celiac disease, ibs, irritable bowel syndrome, prevalence

## Abstract

Background: Many published studies have shown that the prevalence of celiac disease (CD) is higher in individuals with irritable bowel syndrome (IBS); however, the current available evidence is controversial. Moreover, controversy exists regarding the routine screening of CD in IBS patients, as the available results show conflicting evidence.

Objective: This study aimed to determine the prevalence of CD in individuals with IBS presenting at a tertiary care hospital.

Methodology: This one-year cross-sectional study was performed at District Headquarters (DHQ) Teaching Hospital, Gujranwala, Pakistan, between January 2019 and January 2020. A total of 260 persons, who fulfilled the inclusion and exclusion criteria, were selected through a non-probability consecutive sampling technique. Patients having positive anti-tissue transglutaminase (anti-TTG) IgA and IgG antibodies were diagnosed as having probable celiac disease (PCD). Data were collected and compiled using IBM SPSS Statistics for Windows, Version 23.0 (IBM Corp., Armonk, New York, United States). Post-stratification, the chi-squared test was applied, taking p≤0.05 as significant.

Results: The mean age of the patients was 49.4±18.5 years. Gender distribution showed that 168 (64.6%) were males, while 92 (35.4%) were females. Among 260 patients, 39 (15%) were diagnosed with CD. Duration of IBS is associated with increased prevalence of CD, and this association is statistically significant (p<0.001).

Conclusion: Patients with IBS, irrespective of age and gender, are more susceptible to develop CD compared to the general population. Based on these findings, we recommend routine screening for CD with serologic markers in all IBS patients to identify individuals with latent or potential CD.

## Introduction

Irritable bowel syndrome (IBS) is among the most prevalent gastrointestinal functional disorders, affecting up to 3-15% of the Asian population [1]. Characteristic features of IBS are unexplained abdominal pain, uneasiness, and bloating along with alterations in bowel movements. Etiologies of IBS include psychological factors, genetic linkage, and history of recent illness, intestinal bacterial overgrowth, food allergy, alteration in bowel motility and/or secretion, visceral hypersensitivity, change in central nervous system sensory processing, disturbed autonomic nervous system regulation, and serotonin abnormalities [2].

Patients are diagnosed on the basis of the Rome III criteria for the diagnosis of IBS [3]. According to the Rome III criteria, a diagnosis of IBS requires recurrent abdominal pain or discomfort occurring at least three days per month over the past three months, with symptom onset at least six months prior, and the pain must be linked to at least two of the following: relief with defecation, a change in stool frequency, or a change in stool form. Conventional IBS therapy is mainly symptomatic. Various pharmacological agents are used to provide symptomatic relief and include bulking agents, anti-diarrheal drugs, laxatives, anti-spasmodics, antibiotics, and anti-depressants [4]. Non-pharmacological measures like diet and psychotherapy are also used.

Celiac disease (CD) is an autoimmune-mediated enteropathy characterized by small intestinal damage resulting in the loss of absorptive villi and crypt hyperplasia. This culminates in malabsorption syndrome [5]. CD is precipitated by ingestion of gliadin, usually present in gluten of wheat, in sensitized individuals [6]. The prevalence of CD is 1:200 in the Asian population [7]. Clinical symptoms include chronic diarrhea, abdominal distention, and growth retardation. The extraintestinal manifestations include anemia, vitamin D deficiency, dermatologic manifestations, endocrinopathies, infertility, and others. It is associated with other autoimmune diseases like diabetes, Sjögren's syndrome, pernicious anemia, autoimmune hepatitis, and autoimmune hypothyroidism [8].

Jejunal biopsy revealing villous atrophy remains the gold standard for diagnosing CD. Instead of doing a jejunal biopsy, which is cumbersome, a distal duodenal biopsy is preferred, as it is convenient and has comparable results to a jejunal biopsy. Other non-invasive tests include IgA and IgG antibodies against gliadin, endomysium, and tissue transglutaminase (TTG) with a sensitivity and a specificity of 85% and 87%, 76% and 81%, 92% and 98%, and 85% and 97%, respectively [9]. A gluten-free diet is the best current treatment available for CD.

The literature suggests that a strong correlation exists between gluten sensitivity and IBS. It has been postulated that functional symptoms can also result from gluten sensitivity. However, the exact prevalence of CD in IBS patients is unknown, with some studies reporting a low prevalence of 2.79% and 3.2%, while others reported a high prevalence of about 12.5% [10-13]. Moreover, no local studies have been conducted to find out the prevalence of CD in IBS patients. This study aims to assess the prevalence of CD among individuals with IBS in order to strengthen local support for the routine use of anti-TTG antibody screening in the local population.

## Materials and methods

Study sample and design

This one-year cross-sectional study was performed in the Department of Gastroenterology, District Headquarters (DHQ) Teaching Hospital, Gujranwala, Pakistan, from January 2019 to January 2020. The study received approval from the Institutional Review Board of Gujranwala Medical College/DHQ Teaching Hospital under approval number 124/2019/MS.

Inclusion criteria

All individuals aged 18-80 years, irrespective of gender, who fulfilled the Rome III criteria for IBS, presenting at the Department of Gastroenterology at DHQ Teaching Hospital, were included in the study.

Exclusion criteria

All participants who were already on a gluten-free diet were excluded from the study. Moreover, participants with a known history of other malabsorption disorders, e.g., inflammatory bowel disease, giardiasis, etc., were also excluded from the study.

Sample size

The sample size of this study was 260, and the results were calculated with a confidence level of 95%, an error margin of 3%, and an anticipated CD prevalence of 6.5% in IBS patients. The sample was collected using a non-probability consecutive sampling method.

Data collection

A total of 260 participants who fulfilled the study selection criteria and provided informed consent were selected. The demographic characteristics (gender, age, and IBS duration) were collected for all enrolled patients. A detailed history of IBS symptoms and duration of IBS symptoms were recorded. Blood samples were collected, serum was analyzed for anti-TTG antibodies, and subsequently, those with positive anti-TTG antibodies underwent duodenal biopsy to confirm the diagnosis of CD.

Data analysis

The collected data were entered into IBM SPSS Statistics for Windows, Version 23.0 (IBM Corp., Armonk, New York, United States), and analyzed through it. For quantitative variables, like age and duration of IBS, means and standard deviation (SD) were calculated. Categorical variables such as gender and presence of CD were expressed as frequency and percentage. Data were stratified by age, gender, and duration of IBS. After stratification, a chi-squared test with a significance level of <0.05 was used to assess the association of CD with IBS in each stratum separately.

## Results

The general demographic characteristics of the normally distributed population are shown in Table 1. A total of 260 patients fulfilling the inclusion/exclusion criteria were enrolled in the study. Out of the total 260 participants, 168 (64.6%) were males, while 92 (35.4%) were females, with a male-to-female ratio of 1.8:1. The mean age (±SD) of the study participants was 49.4±18.5 years. According to the age distribution (%), 149 (57.3%) participants were in the >45-year age group, followed by 57 (21.9%) in the 31-45-year age group and 54 (20.8%) in the 18-30-year age group. Among the 260 patients, 130 (50%) had a duration of IBS symptoms <5 days, while 130 (50%) patients had a duration of IBS symptoms ≥5 days. Out of the total study population, 39 (15%) were diagnosed with CD (Table 1).

**Table 1:** Demographic characteristics of the study participants (N=260) IBS: irritable bowel syndrome

Characteristic	Category	Frequency (n)	Percentage (%)
Gender	Male	168	64.6
	Female	92	35.4
Age group	18-30 years	54	20.8
	31-45 years	57	21.9
	>45 years	149	57.3
Duration of IBS symptoms	<5 days	130	50
	≥5 days	130	50
Celiac disease status	Yes	39	15
	No	221	85

Table 2 demonstrates the association between gender, age group, duration of IBS, and prevalence of CD. The prevalence of CD was 16.7% in males compared to 12% in females, with a p-value of 0.309, indicating no statistically significant difference between genders. The age group analysis revealed no statistically significant difference in CD prevalence across different age groups (p=0.666). However, the duration of IBS showed a statistically significant association with CD prevalence. The prevalence of CD was 5.4% in individuals with an IBS duration of <5 days (n=130), compared to 24.6% in those with an IBS duration of ≥5 days (n=130), with a p-value of <0.000001. This indicates a highly significant association between a longer duration of IBS and a higher prevalence of CD (Table 2).

**Table 2:** Statistical associations between gender, age group, duration of IBS, and CD prevalence CD: celiac disease; IBS: irritable bowel syndrome

Category	Subcategory	CD: Yes	CD: No	Total	CD prevalence (%)	Chi-squared value	P-value
Gender	Male	28	140	168	16.7	1.035	0.309
	Female	11	81	92	12		
	Total	39	221	260	15	-	-
Age group	18-30 years	6	48	54	11.1	0.809	0.666
	31-45 years	9	48	57	15.8		
	>45 years	24	125	149	16.1		
	Total	39	221	260	15	-	-
Duration of IBS	<5 days	7	123	130	5.4	22.84	<0.000001
	≥5 days	32	98	130	24.6		
	Total	39	221	260	15	-	-

## Discussion

CD, an autoimmune disorder, was historically regarded as a rare disease; however, recent studies have reported a higher prevalence of approximately 1-2% in the general population [14,15]. CD prevalence is observed to be higher in patients with IBS as compared to the general population. Several research have indicated an even greater prevalence of CD among individuals with IBS, ranging from 3% to 15% [16].

According to various studies, the prevalence of CD differs significantly in different geographical areas. For instance, Palsson et al. reported a CD prevalence of 4.7% in IBS patients [17]. In contrast, Rostami Nejad et al. described a prevalence of 11.4% in IBS patients in Tehran [18]. Consistent with these findings, the current study observed a CD prevalence of 15% among patients with IBS.

Domzal-Magrowska et al. conducted a study to find the association between IBS and CD. Their study results showed that a significantly higher proportion of IBS patients exhibit genetic markers predisposing to CD, with approximately 50% having the HLA-DQ2/DQ8 genotype, compared to 20% in the general population. These findings are suggestive of a potential genetic susceptibility between IBS and CD. Notably, genetic marker distribution was not significantly different across the various IBS subtypes [12].

The study conducted by Wahnschaffe et al. revealed a complex association between gluten, immune response, and symptoms of IBS with diarrhea (IBS-D). According to the results of this study, HLA-DQ2 was detected in 35% of IBS-D patients, despite the fact that anti-gliadin and anti-TTG antibodies were normal. Nevertheless, the strict gluten-free diet resulted in the improvement of symptoms in IBS-D patients, suggesting that patients might be suffering from asymptomatic or latent CD [19].

Various studies have demonstrated an increased prevalence of CD in individuals with IBS, with reported prevalence rates ranging from 0.4% to 11.4% [17-22]. Moreover, the association between CD and IBS is particularly stronger in the IBS-D subtype, where rates have been observed as high as 7% [23].

Ford et al. conducted a meta-analysis and suggested that the prevalence of CD in IBS individuals is approximately four times greater than that observed in the general population [13]. However, conflicting evidence exists regarding the association between CD and IBS. For instance, a study by Hin et al., which involved the assessment of endomysial antibodies followed by duodenal biopsy for those with positive results, found no cases of CD among 132 IBS patients [24].

Routine serological testing for CD was considered justifiable in patients with IBS, according to a systematic review by Cash et al., especially in first-degree relatives of patients with CD [25].

However, there are certain limitations to our study. Firstly, as the study was conducted in the South Asian population, the results could not be applicable to non-South Asian individuals due to different HLA genotypes. In order to establish generalizability, further large-scale investigations involving patients with different ethnicities are suggested. Secondly, the prevalence of CD was not assessed in subtypes of IBS: IBS diarrhea, IBS constipation, and IBS mixed. We strongly recommend that further studies be conducted to evaluate the prevalence of CD in IBS subtypes. Thirdly, this is a single-center study; we recommend a large multi-center study to enhance the robustness of our findings. Lastly, we only used anti-TTG for the diagnosis of CD. We recommend using other serologic markers like anti-gliadin, anti-deamidated gliadin peptide antibodies, and also HLA genotyping (HLA-DQ2/DQ8) to diagnose CD patients with negative anti-TTG.

## Conclusions

Patients with IBS, irrespective of age and gender, are more susceptible to develop CD compared to the general population. Based on these findings, we recommend routine screening for CD with serologic markers in all IBS patients to identify individuals with latent or potential CD. Further larger-scale studies are required to establish the association between IBS and CD to develop guidelines in order to decrease the morbidity and burden of disease.
